# Analysis of Factors Affecting 5-ALA Fluorescence Intensity in Visualizing Glial Tumor Cells—Literature Review

**DOI:** 10.3390/ijms23020926

**Published:** 2022-01-15

**Authors:** Marek Mazurek, Dariusz Szczepanek, Anna Orzyłowska, Radosław Rola

**Affiliations:** Chair and Department of Neurosurgery and Pediatric Neurosurgery, Medical University of Lublin, 20-954 Lublin, Poland; dariusz.szczepanek@umlub.pl (D.S.); a.m.orzylowska@gmail.com (A.O.); rola.radoslaw@gmail.com (R.R.)

**Keywords:** 5-aminolevulinic acid, high-grade glioma, glioblastoma, intraoperative navigation

## Abstract

Glial tumors are one of the most common lesions of the central nervous system. Despite the implementation of appropriate treatment, the prognosis is not successful. As shown in the literature, maximal tumor resection is a key element in improving therapeutic outcome. One of the methods to achieve it is the use of fluorescent intraoperative navigation with 5-aminolevulinic acid. Unfortunately, often the level of fluorescence emitted is not satisfactory, resulting in difficulties in the course of surgery. This article summarizes currently available knowledge regarding differences in the level of emitted fluorescence. It may depend on both the histological type and the genetic profile of the tumor, which is reflected in the activity and expression of enzymes involved in the intracellular metabolism of fluorescent dyes, such as PBGD, FECH, UROS, and ALAS. The transport of 5-aminolevulinic acid and its metabolites across the blood–brain barrier and cell membranes mediated by transporters, such as ABCB6 and ABCG2, is also important. Accompanying therapies, such as antiepileptic drugs or steroids, also have an impact on light emission by tumor cells. Accurate determination of the factors influencing the fluorescence of 5-aminolevulinic acid-treated cells may contribute to the improvement of fluorescence navigation in patients with highly malignant gliomas.

## 1. Introduction

Gliomas are among the most common tumors found in neurosurgery. It is estimated that they constitute 30% of all brain tumors and as much as 80% of malignant lesions [1]. The standard of care for malignant gliomas is maximal tumor resection, followed by radio- and chemotherapy [2]. Unfortunately, despite appropriate treatment, these tumors are prone to recurrences and have an unfavorable prognosis [3,4,5]. One of the reasons for this phenomenon is the high migratory ability of glioma cells, which renders the gross total resection (GTR) highly unlikely [6,7]. The analyses performed showed the presence of tumor cells up to 4 cm from macroscopically visible tumor margins [8,9]. An incomplete resection results in higher risk of neoplasm recurrence and poorer effectiveness of adjuvant therapies, such as radio- and chemotherapy [10,11,12,13,14,15]. For this reason, much attention is paid to the improvement of surgical techniques that help to maximize the percentage of achieved GTR. One of them is intraoperative fluorescence navigation that facilitates intraoperative visualization of the neoplastic tissue through the administration of substances that make it fluoresce [16,17].

Currently, a few substances are used as fluorescent indicators in brain tumor surgery. The most commonly used dye is 5-aminolevulinic acid (5-ALA) [18,19,20]. This compound participates in the heme metabolic pathway [21,22,23]. Usually, it is administered orally to patients in the form of an aqueous solution at a dose of 20 mg/kg body weight, about 3 h before the planned surgery [24,25]. Thereafter, in the body it is metabolized to the heme precursor—protoporphyrin IX (PpIX) [25,26,27]. This compound has two unique features that allow it to be used in fluorescence navigation. One of them is the ability to emit a light wave after excitation by blue-violet light with a wavelength of 375–440 nm. This allows for intraoperative imaging of its deposits thanks to the use of an operating microscope with a special set of filters [19,21,23,28,29,30]. The second unique feature of the dye is its selective accumulation in high-grade glioma cells compared with normal brain tissue. This allows the surgeon to determine the likely margins of the operated lesion while the procedure is still ongoing. Data available in the literature indicate high specificity (83.8–93.9%) and sensitivity (73.9–91.4%) presented by 5-aminolevulinic acid [31,32,33,34,35,36,37,38,39,40].

The first studies on the usefulness of 5-ALA in high-grade glioma surgery were carried out in the 1990s [21,22]. Since then, the use of this dye has been repeatedly demonstrated to improve the percentage of GTR achieved in glioblastoma patients by using intraoperative fluorescence navigation [19,24,29,41,42,43,44]. Additionally, as a meta-analysis by Gandhi et al. shows, this also results in progression-free survival and survival rates of the patients [18]. However, the presence of fluorescence and its nature are not the same for all glial tumors. Many times, despite the supply of an appropriate dose of dye in the appropriate time window, no fluorescence is observed after the tumor is visualized, which significantly hinders the course of the procedure. The goal of this study is a literature review on the topic of the factors affecting the mechanism of fluorescence induced by the supply of 5-aminolevulinic acid.

## 2. Intracellular Metabolism of 5-Aminolevulinic Acid

When considering the metabolism of 5-ALA and its potential influence on the strength of fluorescence, one should remember that the cell can derive 5-ALA from two sources. The first one is the exogenous supply. The dye is administered orally to patients; then it is absorbed from the digestive tract and distributed throughout the body. Exogenous 5-ALA is delivered to cells via special transporter proteins. This group includes peptide transporter 1 (PEPT1) and peptide transporter 2 (PEPT2) [45,46]. The other source of the dye is its endogenous fraction, produced from succinyl-CoA and glycine. This process takes place in the mitochondria and is catalyzed by ALA synthase (ALAS) [46,47,48,49]. Then, endogenous 5-ALA is transported to the cytoplasm, where it can undergo further transformations together with the exogenous fraction.

**Figure 1:**
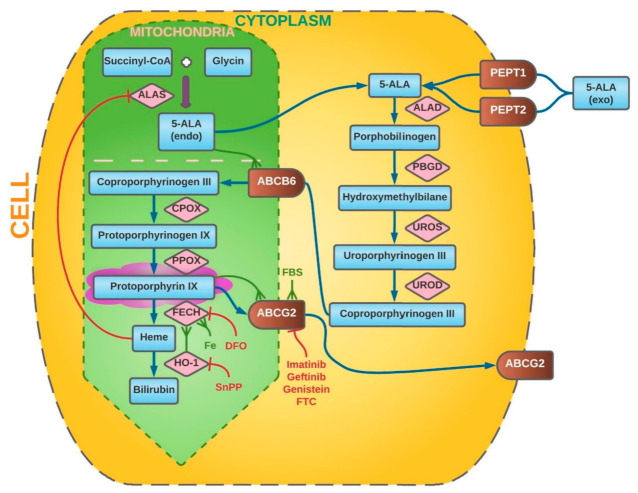
Intracellular metabolism of 5-ALA. The synthesis steps of heme metabolism are labelled by blue arrows (↑). Red indicators (T) point to enzymes and substances that inhibit particular steps of metabolism, whereas green indicators (Ψ) highlight factors that promote them. PEPT 1/2—peptide transporter 1/2, ALAS—ALA synthase, ALAD—ALA dehydratase, PBGD—porphobilinogen deaminase, UROS—uroporphyrinogen III synthase, UROD—uroporphyrinogen III decarboxylase, ABCB6—ATP-binding cassette transporter B6, CPOX—coproporphyrinogen III oxidase, FECH—ferrochelatase, ABCG 2—ATP-binding cassette subfamily G 2 Protein, DFO—deferoxamine mesylate, FBS—fetal bovine serum, SnPP—tin protoporphyrin IX, FTC—fumitremorgin C, 5-ALA (endo)—endogenous 5-ALA, 5-ALA (exo)—exogenous 5-ALA.

The first steps occur in the cytoplasm, while the final transformations take place in the mitochondria [50]. In the first act of metabolism, two 5-ALA molecules condense to porphobilinogen (PBG) in a reaction catalyzed by ALA dehydratase (ALAD). Another name for this enzyme is porphobilinogen synthase (PBGS). Then, porphobilinogen deaminase (PBGD), also known as hydroxymethylbilane synthase (HMBS), catalyzes the fusion reaction of four PBG molecules, resulting in the formation of hydroxymethylbilane (HMB). Its structure is later closed by uroporphyrinogen III synthase (UROS) to form the cyclic uroporphyrinogen III. The next step is its decarboxylation. This reaction is catalyzed by uroporphyrinogen III decarboxylase (UROD), and its product is coproporphyrinogen III. The next stages of metabolism take place already in the mitochondria. The transport of metabolites is mediated by ATP-binding cassette transporter B6 (ABCB6). In mitochondria, coproporphyrinogen III oxidase (CPOX) catalyzes the oxidative decarboxylation of coproporphyrinogen III, resulting in the formation of protoporphyrinogen III. It is further oxidized to protoporphyrinogen IX (PpIX) by protoporphyrinogen III oxidase (PPOX). It is protoporphyrinogen IX that is the main source of fluorescence used in intraoperative 5-ALA navigation. In the last step, Fe^2+^ is included in the pyrrole ring of PpIX. As a result, nonfluorescent heme is formed. This process also takes place in the mitochondria and is catalyzed by ferrochelatase (FECH). Additionally, reaction can be accelerated by heme oxygenase-1 (HO-1) [48,50,51,52,53,54,55,56,57]. If heme and free porphyrin metabolites remain inside the cells for a long time, they can induce oxidative stress and damage them. For this reason, a well-coordinated mechanism of their further transport and degradation is very important [58]. The ATP-binding cassette subfamily G (ABCG) 2 protein [59,60,61] plays a key role in their transmembrane transport. Intracellular metabolism of 5-ALA is shown schematically in Figure 1.

## 3. Alterations in 5-Aminolevulinic Acid Metabolism in Neoplasm Cells

The heme metabolic pathways described above differ between normal and neoplastic cells, resulting in a variation in fluorescence. The reason is both the greater accumulation of PpIX inside the cancer cells and the reduced rate of its transformation. This discrepancy may result from the difference in the rate of division between neoplastic and healthy cells [62,63]. This applies, inter alia, to porphobilinogen deaminase, the activity of which increases during replication [64,65,66]. It was confirmed that the higher activity of this protein concerns rapidly dividing cells, including tumors [64,67,68,69,70,71,72,73]. The increase in PBGD activity may be caused by the administration of 5-ALA itself [74,75]. However, no association has been demonstrated between higher PBGD activity and PpIX accumulation [74,76]. On the other hand, studies on esophageal cancer cells have suggested a correlation between high PBGD activity and decreased FECH function, which could result in increased PpIX accumulation [71]. Further observations did not confirm the existence of this relationship though [64,68,72]. Other enzymes involved in heme metabolic changes are also more active in neoplastic tissues. Greater UROS expression was noted in breast cancer and in tumor biopsies from head and neck cancer patients [73,77]. Another enzyme with altered activity in cancer cells is ferrochelatase. Reduced FECH expression has been demonstrated in many tissues and tumors, including glioblastoma multiforme [64,72,78,79,80]. This condition promotes longer accumulation of PpIX inside cells, which may also be related to the intensity of their fluorescence [78,81,82,83]. However, this phenomenon also applies to other gliomas, including LGG. Studies by Teng et al. showed a decreased level of FECH mRNA expression in glioblastoma, diffuse astrocytoma, and anaplastic astrocytoma cells compared with normal tissue. It is worth noting, however, that the lowest values were for malignant WHO IV tumors [84]. Cancer cells can also be distinguished by the production of endogenous 5-ALA. The key enzyme in this process is ALA synthase (ALAS). Colorectal cancer specimens showed a significantly lower activity of this enzyme compared with normal tissue [78]. However, in the case of lung cancer, the opposite trend was observed [85]. Still, there are no data in the literature regarding ALAS activity in glioblastoma cells.

Neoplastic cells are characterized by differences not only in the activity of enzymes in the hem metabolism pathway but also in the activity of hem transporters and their metabolites. PEPT2, the major protein responsible for transporting 5-ALA into the cell interior, has been shown to be overexpressed in glioblastoma cells [84]. Analogue discrepancy applies to ABCG2, which is also an intermembrane transporter of hem metabolites [86,87,88]. ABCB6 is another transporter belonging to the same family of proteins. Zhao et al. noted that it may also affect the distribution of PpIX. Their studies on glioblastoma cells showed higher expression of this protein in glioma cells compared with normal brain tissue, but the intracellular localization of ABCB6 does not provide unambiguous evidence regarding its influence on the degree of PpIX accumulation [89,90,91,92]. However, the above-presented discrepancies in the metabolism and transport of heme metabolites result in greater accumulation of PpIX in some tumor cells, which is a key aspect used in fluorescence intraoperative navigation [22,93]. Additionally, studies by Stummer et al. in a rat model showed that glioblastoma cells metabolize 5-ALA and collect its excess both in vivo and in vitro, which may interfere with the detection of fluorescence [22]. The heme metabolism is subject to natural regulatory processes. The availability of substrates and intermediates plays an important role in it. An important regulating point is, inter alia, inhibition of ALAS in the feedback mechanism [94]. It can be inhibited directly by the intracellular heme level [95]. However, this compound also influences other stages of metabolism, including the rate of its degradation by heme oxidase [50,83].

However, PpIX fluorescence after exogenous 5-ALA supply is not a common feature of all neoplastic cells. It has been shown many times that it concerns mainly cells exhibiting marked features of malignancy. It is clear that the use of fluorescent intraoperative navigation shows the significant effectiveness of this method in the treatment of patients with high-grade glioma (HGG) [19,24,29,41,42,43,96,97,98,99]. However, for low-grade glioma (LGG), the statistics are not that promising. One of the first reports on the use of 5-ALA in the surgery of low-grade gliomas comes from Ishihara et al. The authors, examining 65 slices from six resected tumors, showed that diffuse astrocytomas exhibit noticeably weaker PpIX fluorescence compared with anaplastic astrocytomas and glioblastoma [100]. This was in line with the later work of Widhalm et al., who showed no light emission for 100% (8/8) of the samples from patients with WHO grade II diffusely infiltrating gliomas [101]. Their later work confirmed these initial observations—of the 215 tumor specimens analyzed from 59 patients with diffusely infiltrating gliomas, only 19% (4/33) of the WHO II tumor samples showed noticeable fluorescence. For comparison, in WHO grade III gliomas, focal PpIX fluorescence was visible in 85% (23/26) of the cases [102]. A similar inconsistency was visible in the insights of Ewelt et al., who detected visible PpIX fluorescence only in 7.7% (1/13) of WHO II glioma and 70.6% (12/17) of higher-grade tumors [32]. Observations on a larger group of patients were conducted by Wadiur et al. The authors analyzed the presence of fluorescence in 110 patients with low-grade glioma tumors in imaging studies and recorded it in 36% (40/110) of the cases. Moreover, subsequent analysis of the results of histopathological examinations revealed that of the WHO II tumors, only 11% (7/65) showed noticeable luminosity. In the case of WHO III and WHO IV tumors, it was 68% (26/38) and 100% (7/7), respectively [103]. Similarly, Jaber et al. in their study proved that of 166 eligible tumors (82 WHO II, 76 WHO III, and 8 WHO IV), PpIX fluorescence was present in more malignant tumors. The authors concluded that if the lesion fluoresces, it is HGG 85% of the time. For LGG, the percentage of glowing tumors was 16% (13/82) [104]. The problem with visualizing pure low-grade glioma fluorescence was also reported by other authors [27,105,106,107,108,109,110]. However, there are case reports of LGG patients showing visible fluorescence [111,112]. Moreover, Marbacher et al. confirmed the presence of detected glow in 40% (8/20) of the analyzed LGGs [108]. Similar value results were shown by Valdés et al. Goryaynov et al. showed even greater fluorescence when out of 27 histologically confirmed tumors (14 diffuse astrocytomas, 6 oligodendrogliomas, 4 pilocytic astrocytomas, 2 gemistocytic astrocytomas, and 1 desmoplastic infantile ganglioglioma), 52% showed fluorescence after exogenous 5-ALA supply at a dose of 20 mg/kg. However, the quality of lighting was diverse—50% of the tumors showed diffuse fluorescence, and 50% focal [113,114]. Some authors suggested that such foci of increased fluorescence in low-grade glioma tissue may indicate local malignancy [114,115].

Interestingly, fluorescence heterogeneity is also frequently observed in tumors with a high proliferation rate. Many have authors shown significant discrepancy in the fluorescence intensity between different glioma cell lines subjected to the same conditions [46,57,116,117]. The phenomenon has also been observed intraoperatively. Stummer et al. noted significant regional heterogeneity in fluorescence intensity for glioblastoma tissue. It has been suggested that the reason for the different intensity of light may be a various cell density within the tumor [101,102,118]. However, it is probably caused by the significant genetic polymorphism of neoplastic cells resulting from the rate of their proliferation. This was very well outlined in a study by Kim et al., in which the authors classified samples from five patients with glioblastoma in terms of luminosity and then subjected them to a thorough RNA sequencing analysis. A total of 585 genes that influence the PpIX accumulation and fluorescence intensity were identified [80]. As previously mentioned, other authors have also suggested a relationship between the rate of cell proliferation and the luminosity [18,19,20]. Additionally, Widhalm et al. analyzed the role of 5-ALA in identifying anaplasia foci in diffusely infiltrating gliomas with nonsignificant contrast enhancement [101]. The results obtained by the authors showed that Ki-67/MIB-1 is significantly higher in the areas of the tumor showing PpIX fluorescence (20% vs. 10%) [101]. In their following work, the authors analyzed features of 215 tumor specimens collected from 59 patients with diffusely infiltrating gliomas with nonsignificant contrast enhancement on MRI. They showed that in fluorescent areas, mitotic rate, cell density, nuclear pleomorphism, and proliferation rate were significantly higher than in nonfluorescing areas. Similar results were presented by Ohba et al., who in their observations on 104 patients with glioma showed that contrast enhancement, WHO malignancy, IDH status, and the Ki-67/MIB-1 index influence intraoperative tumor glow assessed by the surgeon [106]. Likewise, Jaber et al. examined the influence of tumor volume, 18F-FET PET uptake, contrast enhancement, grade, IDH1 mutation status, O6-methylguanine DNA methyltransferase (MGMT) promoter methylation status, 1p/19q codeletion, and Ki-67/MIB-1 and proved that the intensity of fluorescence correlates with the expression of Ki-67 and with the grades of histological malignancy. No similar relationship was found for MGMT status, IDH1 mutation status, or 1p19q codeletion status, however [104]. Equally, Saito et al., on the basis of univariate analysis, also showed a relationship between the intensity of cell proliferation measured with the Ki-67/MIB-1 index and the luminosity induced by 5-ALA supply. It should be noted, though, that a similar relationship also occurred in the case of 1p19q codeletion status in contrast to the previously mentioned work. A work by Ishihara et al. is also worth mentioning, in which on top of the similar relationship between the Ki-67/MIB-1 index and the fluorescence intensity, it was shown that this parameter is also influenced by CD31-microvessel density and larger VEGF expression. However, a more detailed multiple regression analysis showed that only the Ki-67/MIB-1 index was significantly related to the fluorescence intensity [19]. It has been suggested that modifications of tumor metabolism related to increased cell proliferation might affect the activity of enzymes responsible for the intensity of PpIX accumulation [80,119]. Mechanisms affecting the visibility of fluorescence were induced by the supply of 5-aminolevulinic acid.

### 3.1. Blood–Brain Barrier

One of the factors that may influence the accumulation of dyes in cells, and hence the intensity of light, is the structure of the blood–brain barrier (BBB). It is the primary border separating the brain environment from the rest of the body. However, brain tumors can cause dysfunction and degradation, simultaneously affecting tumor growth and the effectiveness of therapeutic strategies. This is also true for fluorescent intraoperative navigation [120]. The potential for 5-ALA penetration into the brain was considered in the past, explaining the presence of neuropsychiatric symptoms in hepatic porphyria [121]. However, observations of the distribution of radiolabeled ALA by Terr and Weiner showed no penetration of the dye into the brain tissue [122]. This was in line with the conclusions of Stummer et al., who did not show the presence of fluorescence caused by 5-ALA administration in patients with a normal blood brain barrier structure [123]. It therefore appears that under normal conditions, it is impermeable to 5-ALA [84]. On the other hand, transport via BBB was noted by McGillion et al., and a slight displacement of 5-ALA within the blood–brain barrier itself has also been shown in other studies [124,125]. Few authors have described the ability of choroid plexus to transport 5-ALA, but it has not been shown that it is able to penetrate the brain tissue [123,126,127]. Therefore, Ennis et al. analyzed the distribution of radiolabeled ALA across the blood–brain and blood–cerebrospinal fluid barrier in rats. The authors showed that in adults, the spread of the dye in the brain tissue was low and suggested that this probably took place by means of passive diffusion since the increase in plasma ALA concentration was not associated with the increase in its distribution to the brain tissue [128]. The lack of active transporters was also consistent with the results of García et al., who analyzed the structure of brain microvessels without proving the presence of transporters enabling the distribution of the dye to the brain tissue [125]. The above observations suggest that despite the possibility of interaction of 5-ALA with membrane transporters in various tissues, the blood–brain barrier in the normal state is practically impermeable to it [129,130,131,132]. Consequently, the condition necessary for the accumulation of PpIX after the exogenous supply of the dye is the state of disturbance of its structure. The severity of BBB dysfunction depends on the location, volume, type, and malignancy of the tumor [133]. In the case of gliomas, the difference in barrier permeability between LGG and HGG has been repeatedly demonstrated. While in tumors of low malignancy BBB disruption is relatively minor, in malignant gliomas it results in edema and the formation of areas with impaired vascular density and integrity [134,135,136,137]. Additionally, astrocytes migrate away from vascular endothelial cells, resulting in the disruption of a barrier structure, thereby affecting BBB permeability [138]. This may provide an explanation for the disparity in PpIX accumulation and fluorescence of lesions depending on their malignancy [28,93,120,134,139].

### 3.2. ABCG2 and ABCB6 Transporters

Intracellular 5-ALA transport may also have an influence on the PpIX accumulation and fluorescence intensity. The ABCG2 transporters, which as previously mentioned are crucial in the distribution of heme metabolites, play an important role in this issue. These are proteins belonging to the larger family of the ATP-binding cassette superfamily located in the plasma membrane [1,61]. Their function is not only to remove porphyrins but also to transport xenobiotics and harmful toxins outside the cell [140]. The presence of ABCG2 was first demonstrated in a study of doxorubicin-resistant breast cancer cells, which gave it its second name, breast cancer resistance protein (BCRP) [141]. Later they were found in many tissues of the body, including the blood–brain barrier, and their overexpression is a characteristic feature of many cancers [30,87,88,142,143,144,145,146,147,148,149]. Moreover, their expression level corresponds to the histological grade of neoplastic lesions [88]. It has been shown that high activity of ABCG2 is associated with a decrease in intracellular accumulation of PpIX after ALA administration, which results in a lower intensity of fluorescence [60,149]. Moreover, the fact that blocking ABCG2 receptors causes the accumulation of porphyrins and ABCG2 blocking with imatinib and gefitinib increases the effectiveness of photodynamic therapy have already been shown [150,151,152,153]. This type of transporter can also be blocked by genistein [154]. Reiner et al. observed the fluorescence intensity in three different GBM cell lines and found no effect of genistein on the intensity of endogenous PpIX glow. However, after 5-ALA was added to the cell lines, there were significant discrepancies due to dye accumulation. The simultaneous application of genistein and 5-ALA increased PpIX fluorescence by 42% for U87MG cells, by 31% for U87wtEGFR cells, and by 54% for U87vIII cells compared with the use of 5-ALA alone [155]. This phenomenon was also observed in studies by Piffaretti et al., which showed the effect of increasing genistein concentrations on the viability of the analyzed glioblastoma cell lines. Moreover, they also noticed an increase in fluorescence of cells incubated in genistein and 5-ALA media compared with 5-ALA alone [46]. A number of other compounds are also known that may affect the expression level and function of ABCG2 transporters, thereby affecting the intensity of PpIX fluorescence, such as Ko143 or flavonoids, including 6-prenylchrysis and tectochrysin [156,157,158,159]. Currently, there are no studies of the impact of their application on the fluorescence intensity in gliomas though. It is worth mentioning that the fluorescence intensity and transport activity can be influenced not only by chemical but also by physical factors. Recent studies on breast cancer stem cells have shown that ultrasound has the effect of reversing chemoresistance by altering the expression of ABCG2 [160]. Interesting conclusions were also brought by a research conducted by Higuchi et al. The authors analyzed the effect of ultrasound on the intensity of fluorescence of cells and on the expression of ABCG2 after 5-ALA administration. It was shown that exposure of the cells to 5-ALA caused a slight increase in the expression of the transporters; however, this effect was suppressed by ultrasounds that reduced the expression of ABCG2. The increase in the intracellular accumulation of PpIX in glioblastoma cells was confirmed by a spectrometer. This applied to all analyzed cell lines, and the effect lasted for over 2 h with some differences in the intensity and dynamics of the increase in glow between different cell populations [161]. It should be mentioned, however, that the inhibition of ABCG2 was not associated with an increase in PpIX accumulation in all observations. Wang et al. used reserpine to lower ABCG2 activity in their study on glioma cancer stem cells (GSCs) and showed that it did not improve the PpIX fluorescence in both the group of GSCs and the control group. Moreover, in the case of GSCs, the effect was even lower accumulation of PpIX [162]. This may be due to the influence of reserpine on other ABC family transporters present in the cell: ABCB6, ABCB7, and ABCB10. Disruption of their function may impair PpIX metabolism, contributing to its lower accumulation in cells [162,163]. However, the abovementioned data show that the regulation of the function of ABCG2 transporters has a great potential to modify PpIX accumulation and tumor cell fluorescence during intraoperative navigation. One should remember yet that long-term administration of ABCG2 inhibitors may be associated with phototoxicity reactions and the disruption of transporters in other parts of the body, such as kidneys, which may have negative consequences for patients [164,165]. Therefore, further observations in this matter are needed in order to develop an optimal strategy for a potential treatment.

Other proteins from the ATP-binding cassette superfamily family may also influence heme transformation processes. In recent years, the role of the ABCB6 protein transporter in the regulation of PpIX metabolism in leukemic cells has been demonstrated [166]. As mentioned before, this protein is responsible for the transport of coproporphyrinogen from the cytoplasm to the mitochondria in order to carry out further changes in the heme synthesis pathway [166]. Zhao et al. in their study analyzed ABCB6 expression in surgical glioma samples and proved a much higher expression of ABCB6 mRNA compared with normal brain tissue. Moreover, this increase correlated with the histological grade of the tumor. WHO IV cells showed the highest expression, but a statistically significant difference was already visible in WHO II cells, compared with normal brain tissue. In the next step, the authors showed that cells with high abundance of ABCB6 were also characterized by higher dye accumulation and higher fluorescence intensity and proved that incubation with exogenous ALA resulted in a further increase in ABCB6 expression [89]. Additionally, cells overexpressing ABCB6 showed a higher level of PpIX accumulation compared with control cells. In the last stage of the study, the authors checked whether inhibition of ABCB6 with a specific siRNA would cause a change in the intensity of dye accumulation. The results showed that ABCB6 expression silencing was associated with a significant decrease in accumulated PpIX in comparison with control cells. The data obtained from the study showed that the above-described phenomena apply only to cells exposed to exogenous 5-ALA [89]. The exact mechanism by which the increase in ABCB6 expression leads to increased PpIX accumulation has not been described to date.

Interestingly, it is not only ABCG2 transporters that can influence the efflux of PpIX from cells. Kitajima et al. conducted a study in which they analyzed the distribution of PpIX in cells derived from the JFCR39 panel. It is a panel consisting 39 human cancer cell lines from nine different tissues (lung, colon, gastric, breast, ovarian, brain, renal, prostate cancer, and melanoma) established by the Japanese Foundation for Cancer Research. The analysis studied the effect of the known ABCG2 inhibitor fumitremorgin C (FTC). In most of the cells of the JFCR39 panel, FTC administration increased the intracellular accumulation of PpIX and decreased its extracellular fraction, which was related to the inhibition of ABCG2 [167]. These results were consistent with the observations of other authors [59,168,169]. Nevertheless, this trend was not true for all of the lines included in the study since some of them showed no strong correlation between the level of dye excretion and ABCG2 expression. Surprisingly, a much stronger correlation was found in the case of the expression of the protein involved in exocytosis, which is dynamin 2. Additionally, inhibition of dynamin 2 significantly increased the accumulation of PpIX by limiting the excretion of the dye [167]. It may be beneficial to perform subsequent observations on glioblastoma cells in order to better understand this mechanism.

### 3.3. Activity of Ferrochelatase (FECH)

Another point of key importance in the accumulation of PpIX is the incorporation of Fe^2+^ into its pyrrole ring, resulting in the formation of nonfluorescent heme. This reaction is catalyzed by ferrochelatase (FECH), a homodimer composed of two amino acid polypeptide chains, located in the mitochondrial membrane [55,78,170,171]. It has been shown that molecular defects or low expression of FECH results in a lower dye content [62,172]. As mentioned earlier, the cells of many neoplasms are characterized by reduced activity of this enzyme [53,80]. This results in a lower conversion rate of PpIX to nonfluorescent heme and hence a higher glow intensity [81,83]. Importantly, the reduction of the activity of FECH may then contribute to an increase in PpIX accumulation inside cells, which has been proven in colorectal and cancer cells. A similar issue, therefore, became the subject of a research by Teng et al. conducted on both human glioma cell lines and surgical specimens. The authors proved that glioblastoma has a prominent downregulation of FECH mRNA expression when compared with normal brain. Other types of gliomas also showed lower albeit less pronounced expression. In addition, inconsistency between different cell lines was also found. The G112 line had the highest expression of ferrochelatase mRNA, while the U87 line had the lowest. This reflected the disproportion in fluorescence after the supply of exogenous 5-ALA with the highest intensity in the case of the U87 line, and the lowest for G112 and SNB19 lines. Moreover, the authors affected FECH activity by siRNA in two cell lines with silencing efficacy greater than 50% for both lines. This resulted in the intracellular accumulation of PpIX and increased intensity of fluorescence in cells exposed to 5-ALA, proving that the use of small RNA interference may allow for a significant increase in the quality of fluorescence achieved [84]. Similar studies on the silencing of the FECH gene were also conducted on other neoplasms [78,84,172,173].

It is worth remembering that other factors may also influence ferrochelatase activity. The natural form of its regulation is the availability of free Fe^2+^ ions [57,63]. Deferoxamine mesylate (DFO) is a compound commonly used in clinical practice for the treatment of cutaneous porphyria. It exerts its effect on blocking FECH activity by chelating iron ions so that they cannot be introduced into the pyrrole ring of PpIX [46,174,175,176]. The result is an increase in the intracellular volume of PpIX and a greater intensity of fluorescence. Hence, an increase in dye accumulation under the influence of DFO has been demonstrated in various neoplastic cells, including glial ones [177,178,179,180,181,182,183]. Reiner et al. analyzed the effect of deferoxamine on three glioblastoma cell lines. The results showed that the supply of DFO in the absence of 5-ALA did not affect the luminance. However, among cells previously exposed to the dye, significant increase in glow intensity was observed in all analyzed cell lines and varied from 6% for U87wtEGFR to 22% for U87vIII lines, respectively, compared with cells treated alone [155]. It was consistent with the observations of other authors. Valdes et al. analyzed the effect of DFO supply in studies conducted on mice implanted with xenograft U251-GFP glioma tumor cells. The animals took a dose of deferoxamine for 3 days and then were given 5-ALA to induce fluorescence. The results showed a 50% increase in PpIX fluorescence intensity in the group of animals receiving DFO compared with the control group. The reported glow level after dye administration was 2.9 times greater than the background in mice treated with DFO, and only 1.9 times greater in the control group [184]. Another interesting conclusion can be drawn from studies by Wang et al. on glioma cancer stem cells (GCSs). The authors showed that the addition of DFO to cells exposed to ALA increased the accumulation of PpIX. This effect was visible both in GCS cells and in the control group [162]. These observations show the great potential of deferoxamine in increasing the fluorescence intensity. However, it should be mentioned that for some authors the results were not as promising. A study by Choudry et al. showed no greater accumulation of PpIX in basal cell carcinoma patients after exposure to DFO [185].

### 3.4. Function of Heme Oxygenase (HO-1)

Another compound involved in the heme homeostasis is heme oxygenase-1 (HO-1), which is responsible for the conversion of heme into biliverdin, carbon monoxide, and Fe^2+^ ions [186,187,188,189]. The high activity of HO-1 results in a large production of Fe^2+^ ions, which, as we mentioned earlier, are the regulating point of FECH efficiency. Moreover, the rapid depletion of heme itself may alter the enzymatic activity in favor of increased PpIX metabolism by FECH. The effect is an increase in the rate of heme synthesis from Fe^2+^ and PpIX, which reduces the intensity of the fluorescence of cells [57,106,162,190,191]. Importantly, this enzyme has been shown to be overexpressed in many conditions that affect the CNS, such as ischemia, brain injuries, or Alzheimer’s disease [192,193,194]. There are also data in the literature showing the upregulation of HO-1 in many neoplasms, such as kidney, prostate, and lung cancer; squamous cell carcinoma of the oral cavity; melanoma; Kaposi’s sarcoma; lymphosarcoma; hepatoma; and chronic myeloid leukemia [195,196,197,198,199,200,201,202,203]. This also applies to brain tumors, including gliomas [204,205]. Moreover, an association was noted between HO-1 expression and tumor progression and its histological grade. Paradoxically, Andaloussi et al. showed that an increase in tumor malignancy is associated with a higher expression of HO-1, which should translate into lower accumulation of PpIX [204,206]. Convergent conclusions were also seen in the work of Gandini et al. The authors showed a significant difference in the expression of HO-1 between glioblastoma and normal brain tissue (54% vs. 22%). The expression level was increased in all analyzed grades and histological subtypes. However, no changes in HO-1 levels were shown with increasing tumor grade. In addition, the authors also noted a significant reduction in survival in patients with WHO grade II and III astrocytoma with high HO-1 expression. It concerned only the cytoplasmic localization of the enzyme [207].

The previously mentioned studies by Wang et al. focused on the observation of enzyme expression in a population of glioma cancer stem cells (GCSs) [162]. These are cells characterized by properties responsible for the initiation of the neoplastic process, often related to the tumor resistance to conventional treatments [208,209]. The observations of Wang et al. showed that the GCSs showed a lower level of fluorescence compared with the cells from the control group following exposure to 5-ALA (34.9% ± 5.4% of the GCSs vs. 68.1% ± 12.6% cells in the control group). When the authors analyzed the level of HO-1 expression, they found that the level of expression of this enzyme in GSCs is high, which results in a lower accumulation of PpIX and, hence, a lower intensity of fluorescence [162]. Data linking high HO-1 expression with decreased PpIX accumulation were provided, inter alia, through studies carried out on melanoma cells. Moreover, the authors showed that enzyme inhibition causes an increase in fluorescence intensity [210]. Similar attempts to enhance the fluorescence of tumor cells by inhibiting the activity of HO-1 were also made in gliomas. The studies conducted by Piffarett and Reiner et al. investigated the effect of tin protoporphyrin IX (SnPP, a synthetic heme analogue with a tin atom in the core) on the intensity of fluorescence induced by 5-ALA supply in glioblastoma cell lines. Due to its structure, SnPP inhibits the activity of HO-1 and is commonly used in pediatric patients suffering from hyperbilirubinemia [211]. Fluorescence intensity analysis showed that the simultaneous use of SnPP and 5-ALA allows for increasing the glow intensity. Moreover, the increase differs between cell lines from 39% for U87wtEGFR to 81% for U87MG. Interestingly, an even greater difference in the light intensity was present with the supply of SnPP alone [46,155]. This relationship may be related to EGFR expression. U87MG cells have normal EGFR expression, while U87wtEGFR overexpresses EGFR, and U87vIII cells express the EGFR version III mutation (EGFRvIII) [46,155].

EGFR, a member of the ErbB receptor family, is an important element in the regulation of cell growth in tissues of epithelial origin [44,212]. Recent studies have shown its key role in tumorigenesis, cell migration, and angiogenesis. Upon binding of a specific ligand, intracellular tyrosine kinase is activated, which leads to the activation of signaling along the Akt, PI3 kinase, and nuclear factor (NF-kβ) proteins pathways. The result is a decrease in cell apoptosis, an increase in proliferation and angiogenesis, and a greater tendency to migrate [2,44,212,213,214]. The mutation present in EGFRvIII cells is characteristic of GBM since 40% of glioblastoma cells have a mutation that overexpresses EGFR, of which about 50% is the EGFRvIII variant, which results in uninterrupted activity of the receptor without the need for external stimulation [215,216,217,218,219,220]. The abovementioned experiments clearly indicate a relationship between the function of HO-1 and the activity of the epidermal growth factor receptor. The presence of a similar relationship has already been observed in non-small cell lung cancer and colon cancer. Studies have shown that EGF stimulation, through NF-kβ activation, contributes to an increase in HO-1 and can be induced by various pathways, such as PI3K, IKK, and protein kinase C (PKC) [221,222,223]. Likewise, Fontana et al. focused their research on the analysis of the influence of EGFR activity on HO-1 function in glioma cell lines: U87MG (low EGFR expression), LN229EGFR (EGFR overexpression), and BS153 (EGFRvIII mutation). Initially, all lines were exposed to 5-ALA, and their fluorescence was checked. BS151 cells were characterized by the weakest intensity of light, which was probably related to the constitutively active EGFRvIII +. In the next step, EGF was added to the samples. The result was a significant decrease in fluorescence for U87MG and LN229EGFR, while no significant change was observed in BS153 culture. This effect was reversed by EGFR-specific siRNA, which reduced protein expression by approximately 80% in U87MG. The authors suggested that this was related to EGFR receptor activation, which resulted in the promotion of HO-1 transcription and expression in a concentration-dependent manner. The mutant BS153 cell receptor remained uninterruptedly active with no effect upon the addition of EGF. To test this theory, the authors inhibited the effect of HO-1 by using SnPP (HO-1 inhibitor) and HO-1-specific siRNA. In both cases, the effect was to restore fluorescence in all cell lines, independent of EGFR expression. Additionally, gefitinib, which is a selective inhibitor of the EGF tyrosine kinase receptor, was added to lines previously exposed to EGF. As a result, the fluorescence was restored in U87MG cells, but no effect was obtained in the case of BS153 [57]. These data clearly indicate a strong relationship between EGFR and HO-1 activity. It also suggests a potential cause of inhomogeneous fluorescence in some tumors composed of cells characterized by intratumoral heterogeneity of EGFR/EGFRvIII, which may make it very difficult to determine the extent of resection [116,224].

### 3.5. Significance of Isocitrate Dehydrogenase (IDH) Status

Other genetic features of tumors may also influence their susceptibility to PpIX accumulation-dependent fluorescence. The new classification of tumors of CNS created by the World Health Organization (WHO), in addition to the standard four-step division according to the degree of histological malignancy, also included a group of neoplasms in which the key role is played by the mutant isocitrate dehydrogenase (IDH) 1/2 [225]. It is widely believed that the IDH1/2 mutation occurs at one of the early stages of gliomagenesis. As a consequence, there are two different pathways of neoplastic cell progression depending on the mutation status [226]. Some authors have even suggested that these discrepancies in the origin of the cells may also affect their ability to fluoresce. It should be noted that, physiologically, isocitrate dehydrogenase 1 is one of the enzymes involved in the Krebs cycle. Its role is to catalyze the oxidative decarboxylation of isocitrate to α-ketoglutarate (α-KG) with simultaneous conversion of NADP (+) to NADPH in the cytoplasm and peroxisomes [227,228]. As mentioned earlier, endogenous ALA synthesis depends on the availability of glycine and succinyl-coenzyme A being the reactants [46,47,48,49,229,230]. This indicates a possible role for IDH in regulating PpIX metabolism. IDH1 mutations are present in 55% of WHO III gliomas and 6% of WHO IV [231]. Their presence causes a decrease in the physiological activity of the enzyme, with the simultaneous production of R-2-hydroxyglutarate (2-HG) through the consumption of NADPH, which is an oncometabolite favoring neoplastic transformation [232,233,234,235].

In their research, Ohba et al. analyzed 104 patients operated on for glioma for factors that could affect the fluorescence of the tumor tissue exposed to 5-ALA. Analysis of the collected data showed that among glial tumors, cells with the IDH1/2 mutation showed less fluorescence compared with cells without this mutation. Interestingly, this relationship concerned both high- and low-grade glial tumors. Additionally, in order to find out about the nature of this phenomenon, the authors analyzed the glow intensity of two glioma cell lines in vitro: NHAE6E7hTERTIDH1mut, which was transformed by mutant IDH1, and NHAE6E7hTERTRas, which was transformed by H-Ras (wild-type IDH1 model). Both lines were exposed to 5-ALA. Then, the accumulation of PpIX in both cell types was assessed. The authors showed that the concentration of the dye was lower in NHAE6E7hTERTIDH1mut than in NHAE6E7hTERTRas. This confirmed the genetic basis for the difference in cell fluorescence depending on the IDH1/2 mutation status. The authors suggested that a potential reason for this relationship is the greater activity of FECH and HO-1 in mutant cells, which results in an increase in PpIX metabolism and its lower accumulation [106]. Similar conclusions were drawn by Hickman et al. They analyzed 58 patients with HGG operated under the guidance of intraoperative fluorescence navigation using 5-ALA. The results showed a statistically significant predominance of tumors with an IDH mutation in the group of nonfluorescent lesions—70.6% of tumors with and 100.0% without IDH mutation showed PpIX fluorescence [236]. The presented data were consistent with the results of other authors. In their observations on 60 patients with astrocytic or oligodendroglial tumors, Saito et al. studied the effect of IDH1 status, 1p19q loss of heterozygosity (LOH), the MIB-1 labelling index, the tumor margin, heterogeneity, and contrast enhancement on MRI scans. The authors showed that only the status of isocitrate dehydrogenase 1 allowed for predicting the fluorescence of the tested cells to a statistically significant degree. Their data indicated that only 15% of the cells with the IDH1 mutation showed intraoperative florescence induced by 5-ALA administration [109]. On the other hand, Jaber et al., who also studied the factors influencing the fluorescence of cells after 5-ALA administration, did not show any correlation between the intensity of fluorescence and IDH1, 1p/19q, and MGMT promoter methylation status. It should be noted, however, that the study was deliberately selected for glial tumors not showing radiological features characteristic of glioblastoma multiforme. Out of 166 samples, postoperative histopathological examination revealed 82 WHO II, 76 WHO III, and 8 WHO IV tumors [104].

The role of IDH1 in PpIX metabolism was also investigated by Kim et al., whose cohort included tumor samples from 5-ALA fluorescence-guided surgeries in 35 patients with WHO III gliomas. Postoperative examination of tumor samples revealed the presence of IDH1 mutations in 24 of them, of which 16 showed luminosity. The authors showed a statistically significant relationship between the presence of a mutation and tumor fluorescence. In the next stage of the experiment, PpIX analysis was performed in glioblastoma cell lines. The lines U87MG-IDH1WT harboring wild-type IDH1 and U87MG-IDH1R132H representing the mutant gene variant were used in the experiment. The authors showed a significant delay in PpIX metabolism due to the IDH mutation. In the case of U87MG-IDH1WT cells, an intense increase in fluorescence was noted as early as 1 h after incubation. In U87MG-IDH1R132H cells, the increase in fluorescence was noticeably later. In order to understand the exact nature of this relationship, the authors assessed tricarboxylic acid (TCA) cycle-related metabolite changes using LC–MS. After administration of 5-ALA, a significant increase in the concentration of citrate and a decrease in the concentration of α-ketoglutarate (α-KG) in cells presenting the mutated gene variant were noted. A similar relationship was not observed in the U87MG-IDH1WT line. Additionally, a higher production of R-2-hydroxyglutarate (2-HG) was demonstrated in mutant cells both with and without exposure to 5-ALA. The authors also observed a decrease in baseline NADPH levels in cells bearing the IDH1 mutation compared with WT. After, exposure to 5-ALA NADPH was almost depleted in both cell lines [218]. It has therefore been hypothesized that it is the balance of this compound that may be crucial for heme metabolism and PpIX accumulation.

NADPH is involved in many metabolic chains of the organism and plays an important role in neoplastic cells in which the pathways of its transformation are often disturbed [237,238]. As mentioned before, physiologically, ALA is produced from the Krebs cycle succinyl-CoA and glycine in a reaction catalyzed by ALA synthase [45,93,190,230]. This enzyme is the natural regulatory point of heme metabolism. The excess of heme inhibits the synthase function (inhibiting endogenous ALA production) and at the same time stimulates the action of HO-1, which in the feedback mechanism enhances its degradation [230]. HO-1 breaks down heme together with NADPH reductase. It has been shown that HO-1 activity can be increased also in the presence of NADPH and NADH [239]. The presence of the IDH1/2 mutation greatly influences the availability of NADPH. Data available in the literature suggest that its primary source in human brain cells and gliomas is the pentose phosphate pathway regulated by the activity of isocitrate dehydrogenase, which catalyzes the conversion of isocitrate to α-KG [240]. The IDH1/2 mutation causes a decrease in enzyme activity, leading to impaired NADPH production. In addition, its consumption is also increasing in the pathological production of 2-HG from a-KG in the reduction process dependent on NADPH [233]. The effect may be a significant deficiency of NADPH, resulting in an insufficiently effective work of HO-1. It has been shown that in glioblastoma cells with an IDH1 mutation, the production of the compound is reduced by more than 40% [235,241]. The addition of exogenous ALA overactivates the heme metabolic pathway to break down 5-ALA excess. With concomitant NADPH deficiency, HO-1 function is limited, resulting in low FECH expression and consequently increased PpIX accumulation. It is this mechanism that likely causes the temporary increase in fluorescence in IDH-1 mutant cells demonstrated by Kim et al. [218].

**Figure 2:**
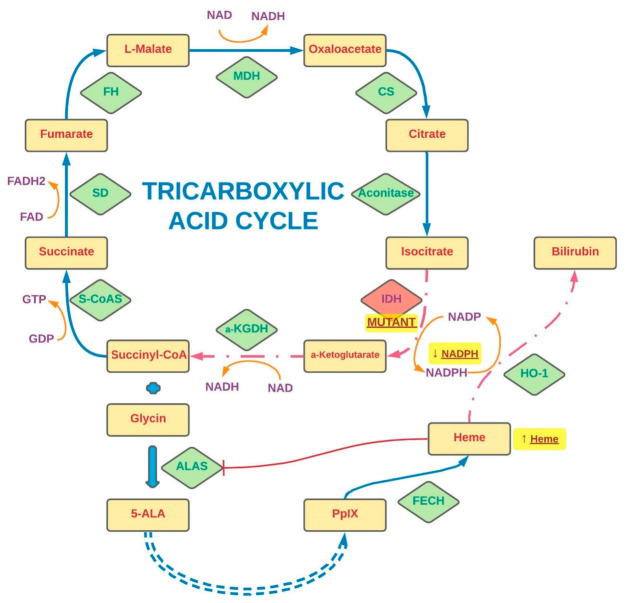
The stages of the tricarboxylic acid cycle and the effects of IDH mutations on protoporphyrin IX metabolism. The synthesis steps are indicated by solid arrows, and the regulatory steps by dashed arrows. The shortened conversions are marked by a double-dashed arrow. A red indicator (T) points to a transformation inhibited by another factor. The effects of IDH mutations are highlighted by a yellow glow. S-CoAS—Succinyl-CoA synthetase, SD—succinate dehydrogenase, FH—fumarase, MDH—malate dehydrogenase, CS—citrate synthase, IDH—isocitrate dehydrogenase, α-KGDH—α-ketoglutarate dehydrogenase, ALAS—ALA synthase, FECH—ferrochelatase, HO-1—heme oxygenase-1, 5-ALA—5-aminolevulinic acid, PpIX—protoporphyrin IX, NAD—nicotinamide adenine dinucleotide, NADP—nicotinamide adenine dinucleotide phosphate, GDP—guanosine diphosphate, GTP—guanosine-5′-triphosphate.

The authors explored the role of NADPH in the regulation of PpIX accumulation and fluorescence with another paper in which three types of glioblastoma tissue (collected from five patients) were carefully scrutinized and characterized in terms of luminosity as strong, weak, and absent. Then, using RNA sequencing, they showed that the expression of 77 genes was directly and that of 508 genes inversely proportional to the intensity of the fluorescence. The effects of IDH mutations on protoporphyrin IX metabolism are shown in Figure 2. In the next stage of the experiment, Kim et al. examined the role of glutaminase 2 (GLS2) in the heterogeneity of the tumor tissue fluorescence [80]. Glutaminase is an enzyme that converts glutamine to glutamate and ammonium in the mitochondria [242]. Importantly, it has been proven that glutamine is the basic metabolic fuel for neoplastic cells, including glial ones [80,119,243,244,245]. The glutamate derived from the transformation is then converted to α-KG and incorporated into the Krebs cycle [242]. Glutaminase has two isoforms: the renal type (GLS), which is expressed in most tissues, and the hepatic type (GLS2), which is present in the brain, liver, and pancreas [246]. They differ in terms of regulating factors and their role in the oncogenesis process [235,242,244,247,248]. In glioblastoma cells, silencing of GLS induces apoptosis, while overexpression of GLS2 inhibits tumor growth [249]. An experiment by Kim et al. showed that GLS2 expression inversely correlated with the intensity of cell fluorescence. Likewise, the transfection of the GLS2 gene construct into the glioblastoma cell lines (T98G, U87MG, and LN18) resulted in a decrease in PpIX accumulation and lower glow level in the samples. It was thus confirmed that decreased GLS2 expression is associated with increased PpIX fluorescence intensity [80]. Importantly, downregulation of GLS2 has been recognized as a hallmark of glioma cells [244,245,248,250]. Conversely, Kim et al. in their study showed that high GLS2 expression was associated with an increase in NADPH production. While exposure to 5-ALA results in a significant reduction in NADPH/NADP and reduced glutathione (GSH)/oxidized glutathione (GSSG) levels, GLS2 expression partially reverses this effect, for it is associated with an increase in NADPH/NADP levels [80]. The collected data show that cells with high GLS2 expression are characterized by a high ability to metabolize 5-ALA, while the reduction of its expression (characteristic of gliomas) may contribute to a delay in metabolism and, as a result, greater accumulation of PpIX, inducing a transient increase in cell fluorescence [80,218]. In order to confirm this relationship, Kim et al. checked the NADPH/NADP ratio in glioblastoma areas characterized by a different light intensity. It has been shown that the nonfluorescent regions show an increased NADPH/NADP ratio compared with the positive glow regions [80].

## 4. Effect of Accompanying Treatment on 5-Aminolevulinic Acid-Induced Fluorescence

Discrepancies between the results of in vitro studies and the actual fluorescence observed clinically during surgery might also raise suspicions of potential disturbances in PpIX metabolism induced by concomitant treatment administered to patients with gliomas. Accordingly, epileptic seizures are a very common symptom associated with intracranial tumors. It is estimated that they occur in over 40% of patients with HGG [251]. The proportion increases even more in recurrent lesions [252]. For this reason, the use of antiepileptic drugs (AEDs) is very common in this group of patients [253,254,255]. Standard treatment options include phenytoin (PHY), carbamazepine, valproic acid, and levetiracetam (LEV) [256,257,258]. Some authors have suggested that the administration of antiepileptic drugs may interfere with the PpIX accumulation and fluorescence process induced by 5-ALA administration. This relationship was demonstrated in a previously mentioned study performed on LGG by Goryaynov et al. The authors showed that among the group of patients taking antiepileptic drugs, tumor fluorescence was seen in only 27% of cases. In comparison, for those without this form of treatment, the percentage was 83% [114]. In another study, Lawrence et al. used the glioblastoma cell line (U87MG) exposing the cells to antiepileptic drugs (phenytoin, valproic acid, and levetiracetam) as well as other substances commonly used in patients with intracranial tumors, such as steroids (dexamethasone) and antidepressants (desipramine). The results showed that for all these substances, except levetiracetam, a reduction in PpIX synthesis in GBM cells was present. What is more, combining dexamethasone with any of the drugs (including levetiracetam) resulted in an even greater reduction in a dye production. It is worth noting, however, that the supply of the steroid was associated with greater PpIX cell retention compared with the control sample. Concomitant use of dexamethasone with desipramine, valproic acid, or levetiracetam did not affect dye retention, while the combination of the steroid with phenytoin was associated with its significant reduction [259]. It has also been suggested that the supply of corticosteroids may seal off the blood–brain barrier, leading to weaker 5-ALA penetration into tumor cells and subsequently weaker PpIX fluorescence [84,260]. Interestingly, one of the first studies on intraoperative navigation using 5-ALA considered including preoperative dexamethasone in the standard operating protocol [24,261]. However, this is not a common practice at present in view of the available data. In another study, Hefti et al. analyzed the effect of phenytoin and levetiracetam on the accumulation of PpIX in glioblastoma cell lines (U373 MG and U-87 MG) and in tumor samples obtained from biopsies of patients who received 5-ALA. The authors demonstrated a dose-dependent reduction in PpIX accumulation in all cell types exposed to phenytoin. Interestingly, no similar relationship was found for levetiracetam [262]. Since the authors related this phenomenon to the disturbance of the PpIX mitochondrial synthesis, they assessed their function by measuring the mitochondrial membrane potential (MMP). Again, the study showed a reduction in mitochondrial membrane potentials only after exposure to phenytoin. At the same time, no morphological and necrotic changes or disturbances in glutathione status, being an indicator of oxidative stress, were observed for both drugs [218]. It was suggested that the changes observed were consequences of the damage to protein and lipids induced by phenytoin and its metabolites, resulting in membrane disorders [262,263,264]. To conclude from studies by Hefti et al. and Lawrence et al., one ought to remember that in patients operated with the use of 5-ALA for fluorescent intraoperative navigation, levetiracetam should be the preferred antiepileptic drug [259,262].

Still the exact mechanism of the influence of concomitant pharmacotherapy on PpIX metabolism remains unknown. Haust et al. in 1989 described the effects of antiepileptic drugs (carbamazepine and valproic acid) on 5-aminolevulinic acid dehydratase and uroporphyrinogen I synthetase activities in erythrocytes of a vitamin B6-deficient epileptic boy. The authors reported that long-term drug use led to decreased activities of 5-aminolevulinic acid dehydratase and uroporphyrinogen I synthetase, while increasing the concentration of erythrocyte protoporphyrin. Pb poisoning, iron depletion, and erythropoietic protoporphyria [265] were not observed. This suggests the direct influence of antiepileptic drugs on the PpIX synthesis enzymes in the mitochondria; however, a detailed understanding of this relationship requires further observations. Not all authors have agreed about the effects of AEDs and steroids on PpIX accumulation. Wadiura et al., who retrospectively analyzed the treatment of 110 glioblastoma patients (WHO II–IV) operated on with 5-ALA navigation, found that visible fluorescence was noted only in 35% of the patients. Nonetheless, the majority of the group consisted of low-grade tumors (WHO II—59%). Of all patients, 66% of the group received preoperative premedication with antiepileptic drugs. The most frequently administered drug was levetiracetam alone or in combination with substances from other groups. Analysis of the collected data showed no statistically significant correlation of the percentages of 5-ALA fluorescence between the groups of patients not taking AEDs (29%), taking LEV alone (43%), taking LEV with another AED (45%), and taking other AED (32%). Still, some of the patients enrolled in the study were also premedicated with dexamethasone (24%). In those cases, univariate analysis showed a statistically significant difference in the percentages of tumors with positive fluorescence between patients receiving preoperative steroid therapy (54%) and those without such therapy (31%). It should be noted though that steroids were administered much more frequently in HGG, which may have biased the data to some extent. Comparison of data only for tumors with the same WHO grading did not show a statistically significant relationship in the fluorescence of tumors in patients receiving and not receiving dexamethasone (WHO II: 11% vs. 11%, WHO III/IV: 77% vs. 71% [103].

## 5. Conclusions

According to the data available in the literature, the use of 5-ALA in intraoperative fluorescence navigation may bring notable benefits in the surgical treatment of patients with glial tumors, resulting in a better therapeutic outcome [18,42,43]. Unfortunately, as clinical practice shows, in many cases the operated tumors differ in the level of emitted fluorescence, which often complicates the course of resection. Moreover, this phenomenon remains largely unpredictable, introducing an element of randomness to the therapy of patients. For this reason, it is important to identify factors that may enhance or disrupt the phenomenon of PpIX fluorescence in glial tumor cells exposed to 5-ALA. In the light of the reviewed publications, many authors suggest that these discrepancies may be due to the different expression and activity of enzymes involved in the metabolism of 5-ALA depending on the histopathological characteristics of the tumor. However, as some of the studies show, the different rate of cell division may also have a significant impact on the expression of particular enzymes involved in the metabolism of protoporphyrin IX, such as PBGD, FECH, UROS, and ALAS [64,66,77,78]. In addition, the dynamics of these processes depends also on the function of proteins involved in the transport of dyes and their metabolites. These include ABCB6 and ABCG2 [89,149,153]. The differences in the intensity of the emitted light between different tumors may also result from impaired penetration of the dye through the blood–brain barrier. Most researchers agree that some degree of damage to the BBB by the tumor is a necessary prerequisite for selective 5-ALA accumulation in the tumor cells. This may be one of the differences observed in the intensity of fluorescence or lack of fluorescence in different brain tumors [122,125].

The use of substances and physical methods that potentiate the emitted fluorescence may also be of great significance. Some authors have attempted to identify tumor profiles that favor fluorescence. These studies include the Ki-67/MIB-1 index, MGMT status, IDH mutation, and 1p/19q codeletion. While in the case of the Ki-67/MIB-1 index and IDH mutation data available in the literature are mostly consistent, in other cases, further observations are needed to assess their significance [19,101,106]. Moreover, the drugs used in standard therapy for patients with CNS tumors may also affect 5-ALA metabolism pathways. Some authors have suggested the importance of certain antiepileptic drugs, such as phenytoin, carbamazepine, valproic acid, and levetiracetam, in this regard [262]. This effect was potentiated by the addition of dexamethasone [259]. The mechanism underlying this relationship is still not fully understood. It can take place both through the influence of drugs on the mitochondrial membrane potential and through the influence on the activity of some enzymes, such as UROS or ALAD. This draws attention to the possibility of an appropriate treatment setting in patients for whom intraoperative fluorescence navigation is used.

Furthermore, it is worth noting that the nature of tumor cell fluorescence is a complex phenomenon. This article focuses primarily on variations resulting from different accumulations of PpIX, the main source of excited light. This is a key aspect, especially in in vitro observations, where light measurement is always performed under analogous conditions using the same instruments. However, it should be noted that the fluorescence intensity also depends on both the equipment used to induce PpIX to emit fluorescence (operating microscope, set of filters determining the light wavelength, or characteristics of the lamps used in the laboratory) and the sensitivity of the detection device. The relationship between the fluorescence intensity of PpIX and the excitation light source is described in a paper by Kamp et al. [266,267]. Belykh et al. also conducted a detailed analysis of the light profile of clinical-grade operating microscopes used for PpIX visualization [268]. In the case of in vivo studies, photobleaching is also an issue. This phenomenon consists in a gradual reduction of the fluorescence emitted by PpIX under prolonged exposure to excitation light [18,268]. It should be mentioned that in some of the works describing intraoperative light emission in vivo in which it was impossible to use specialized measuring equipment, the assessment was made on the basis of the surgeon’s judgement, which unfortunately is an imperfect tool. This highlights the problem of the difficulty in objectively assessing the intensity of emitted fluorescence. However, some authors have made efforts to develop a tool that allows more accurate and reproducible measurements [268]. The above article summarizes the currently available knowledge regarding the differences in the level of fluorescence emitted among the data available in the literature. However, in order to fully realize the potential of this therapeutic method, it is crucial to understand all the relationships governing protoporphyrin IX metabolism and their influence on fluorescence emission by tumor cells. This will allow both for increasing the effectiveness of this navigation method and for defining the optimal group of patients in whom the use of fluorescence navigation can bring maximum benefit.

## Data Availability

Not applicable.
